# Reconstruction of Perineal Defects: A Comparison of the Myocutaneous Gracilis and the Gluteal Fold Flap in Interdisciplinary Anorectal Tumor Resection

**DOI:** 10.3389/fonc.2020.00668

**Published:** 2020-05-06

**Authors:** Jan R. Thiele, Janick Weber, Hannes P. Neeff, Philipp Manegold, Stefan Fichtner-Feigl, G. B. Stark, Steffen U. Eisenhardt

**Affiliations:** ^1^Department of Plastic and Hand Surgery, Medical Center, University of Freiburg, Freiburg, Germany; ^2^Department of General and Visceral Surgery, Medical Center, University of Freiburg, Freiburg, Germany

**Keywords:** reconstructive surgery, gracilis flap, gluteal fold flap, perineal defect, anorectal tumors

## Abstract

**Introduction:** Resection of anorectal malignancies may result in extensive perineal/pelvic defects that require an interdisciplinary surgical approach involving reconstructive surgery. The myocutaneous gracilis flap (MGF) and the gluteal fold flap (GFF) are common options for defect coverage in this area. Here we report our experience with the MGF/GFF and compare the outcome regarding clinical key parameters.

**Methods:** In a retrospective chart review, we collected data from the Department of Plastic Surgery of the University of Freiburg from December 2008–18 focusing on epidemiological, oncological, and therapy-related data including comorbidities (ASA Classification) and peri-/postoperative complications (Clavien-Dindo-System).

**Results:** Twenty-nine patients were included with a mean follow-up of 17 months. Of the cases, 19 (65.5%) presented with recurrent disease, 21 (72.4%) received radiochemotherapy preoperatively, 2 (6.9%) received chemotherapy alone. Microscopic tumor free margins were achieved in 25 cases (86.2%). 17 patients (7 men, 10 women, rectal adenocarcinoma *n* = 11; anal squamous cell carcinoma *n* = 6; mean age 58.5 ± 10.68, mean BMI 23.1, mean ASA score 2.8) received a MGF (unilateral *n* = 10; bilateral *n* = 7). Twelve patients (7 men, 5 women, rectal adenocarcinoma *n* = 7; anal squamous cell carcinoma *n* = 4, proctodeal gland carcinoma *n* = 1, mean age 66.2 ± 9.2, mean BMI 23.6, mean ASA score 2.6) received coverage with a GFF (unilateral *n* = 4; bilateral *n* = 8). Mean operation time of coverage was 105 ± 9 min for unilateral and 163 ± 11 for bilateral MGFs, 70 ± 13 min for unilateral and 107 ± 14 for bilateral GFFs. Complications affected 62%. There was no significant difference in the complication rate between the MGF- and GFF-group. Complications were mainly wound healing disorders that did not extend the hospital stay. No flap loss and no complication that lead to long-lasting disability was documented (both groups). Pain-free sitting took more time in the GFF-group due to the location of the donor site.

**Conclusion:** MG-flaps and GF-flaps prove to be reliable and robust techniques for perineal/pelvic reconstruction. Though flap elevation is significantly faster for GF-flaps, preoperative planning and intraoperative Doppler confirmation are advisable. With comparable complication rates, we suggest a decision-making based on distribution of adipose tissue for dead space obliteration, intraoperative patient positioning, and perforator vessel quality/distribution.

## Introduction

Surgical treatment of rectal and anal diseases may result in perineal defects that affect the surface and lead to loss of volume in the lesser pelvis, following abdominoperineal resection of the rectum (APR) or pelvic exenteration (PE) (1). The vertical rectus abdominis myocutaneous (VRAM) flap is a commonly used reconstructive option and widely reported in the literature (2–4). However, abdominal myocutaneous flaps may be unavailable because of pre-existing abdominal scars, the need for colostomy/urostomy or unacceptable abdominal wall sequelae (2, 5–7). The surgeon is therefore required to consider alternative reconstructive strategies that should involve the following: (1) Provision of a flap with a safe vascularization as recruitment of well-vascularized tissue into a complex wound is crucial and main parts of the flap will not be accessible for perfusion monitoring. (2) Dead space obliteration to prevent the risk of intestinal prolapse, which depends on tumor dimension and location. (3) Accessibility of the donor site, which depends on patient positioning for oncosurgery to minimize the operation time. (4) Keeping donor site morbidity to a minimum, as patients present with significant comorbidity, preoperative radio- and chemotherapy, and a high risk for wound complications (8).

Numerous alternative techniques to the VRAM flap have been described, predominantly using abdominal, pudendal, gluteal, and thigh donor sites (2, 9, 10). Among those, is the myocutaneous gracilis flap (MGF), a well-described alternative to the VRAM flap for genital and perineal reconstruction (11, 12). Functional donor site morbidity of the MGF is advantageous and flap elevation can easily be performed. Recently, perforator based local flaps of the perineal and gluteal region have been introduced in perineal coverage. One of those is the fasciocutaneous gluteal fold flap (GFF) that is based on perforators of the internal pudendal artery (13). First described by Yii and Niranjan in 1996, the flap has been well-described in vulval and vaginal reconstruction and gradually gains popularity for perineal defect coverage (14, 15). Here we present our experience of using the MGF and the GFF uni- and bilaterally for reconstructing perineal defects after resection of anorectal malignancies and compare the outcome regarding clinical key parameters.

## Patients and Methods

### Data Collection

In a retrospective chart analysis, we evaluated all patients that underwent APR or PE at our institution referred for plastic surgery closure between December 2008 and 2018. Data were categorized as demographic, therapy-related, or outcome-related. Patients with the need for vaginal wall reconstruction and patients that received a VRAM flap for defect coverage were excluded. Demographic data included age at the time of surgery, gender, body mass index (BMI), and concomitant diseases. The latter were summarized using the American Society of Anesthesiologists Physical Status Classification System (ASA), a six-point scale to measure the patients' preoperative global health (16). Oncosurgical data consisted of tumor histology, stage of disease, presentation status (primary or recurrent disease), the initial oncological treatment performed (radiotherapy and chemotherapy), the oncosurgical resection procedure (APR or PE), and the achieved resection margins (R0, R1, R2). Reconstructive data included the flap type (MGF or GFF, unilateral or bilateral) and operation time for defect coverage. Postoperative outcome data included all complications or adverse events occurring within 30 days of the operation (classified according to the Clavien-Dindo system), hospital stay, complications that were seen in the period 30 days after the operation until the last follow-up with the potential for long-term disability, and last follow-up. Oncological outcome data such as recurrent disease, distant metastasis and survival status were not included in the study. Informed consent and approval for the publication of photographs were obtained from the patients. The study was approved by the University of Freiburg Ethics Committee, Germany (approval number 357/19). The design and performance of the study are in accordance with the Declaration of Helsinki.

### Statistical Analysis

Analyses of data were performed with GraphPad Prism version 5.0 software (GraphPad Software, San Diego, CA). For comparison of 2 groups, a 2-tailed *t*-test was used. Surgical complications of different grades were analyzed in a 2-way repeated-measure ANOVA. Contingency tables were analyzed by the fisher's exact test. All groups and prognostic factors (gender, age, BMI, comorbidities, preoperative radiotherapy, preoperative chemotherapy, primary disease, recurrent disease, number of flaps, and complications) were analyzed by univariate analysis. A *p* < 0.05 was considered statistically significant.

### Surgical Technique

#### Myocutaneous Gracilis Flap

With the patient in frog leg position, the adductor longus muscle was palpated and a line was marked right behind the adductor longus along the axis of the gracilis muscle on both sides. A mark was made 1 hand-breadth below the inguinal crease, which approximates the location of the primary vascular pedicle (5). Following oncosurgical resection, the left thigh was addressed preferentially in case of a right-handed surgeon. The skin paddle was outlined over the muscle and over the posterior edge of the adductor longus muscle where the intermuscular septum is located and carries vessels to supply the overlying skin. The lengths of the skin paddle can safely comprise the proximal two-thirds of the underlying muscle. Regarding width, the “pinch test” allowed for direct donor site closure; in our patients, about 7 cm could be safely closed primarily. A skin bridge was left between the locations of skin the island and the perineal defect. Preparation of the flap was started from distally, in order to confirm the gracilis muscle and locate its skin territory. Afterwards, the muscle fascia of the adductor longus muscle was exposed via the anterior incision. The fascia was incised and elevated posteriorly in order to incorporate and protect the intermuscular septum. The main vascular pedicle was visualized and freed from its surrounding tissue to the end. Branches to the adductor longus muscle were thereby identified, clipped, and divided. The obturator nerve to the gracilis was identified and divided. The gracilis muscle was freed from the surrounding tissue. Sutures between the muscle and its skin paddle prevented tension forces to the perforators. The flap was then cut distally and tunneled into the defect. Dead space obliteration was evaluated with the colorectal surgeon. In cases of insufficiency, the right sided flap was elevated, and in most cases deepithelialized and buried. The donor site was closed primarily.

#### Gluteal Fold Flap

With the patient in the standing position, the gluteal fold was marked (2). The patient was then put in the lithotomy position to identify the pudendal artery perforators along the medial pole of the gluteal fold using a hand-held Doppler probe or color duplex imaging in the region of the ischial tuberosity on both sides. Following the oncosurgical resection, the perforators were reevaluated intraoperatively. In case of a satisfying distribution and signal, the flap dimensions were outlined, centered on the gluteal fold, and extending for 3–4 cm on either side of it, depending on the “pinch” (to allow direct donor site closure) and ensuring an adequate size to cover the anticipated perineal defect (2). The flap was then raised along a subfascial plane under careful preservation of the perforators through intra-operative Doppler assistance. In this respect, the fibrofatty tissue of ischiorectal fossa was preserved, as it contains the rich network of perforators of the internal pudendal artery and the accompanying vein (13). Skeletonization of the perforators was avoided. The flap was then transposed into the defect as a propeller flap (Type I-1 propeller flap according to Hashimoto et al.) as this allowed a wider arc of rotation than a type I-2 transposition flap (17). The sufficiency of dead space obliteration was re-evaluated with the colorectal surgeon, resulting in uni- or bilateral flap elevation. In cases of bilateral coverage, one flap was deepithelialized and buried. Inset was without tension and the donor site was closed primarily.

## Results

In a 10 years period, 24 myocutaneous gracilis flaps (unilateral MGF *n* = 10; bilateral *n* = 7) and 20 gluteal fold flaps (unilateral *n* = 4; bilateral *n* = 8) were performed for perineal defects following anorectal tumor excision in 29 patients. Fifteen out of 29 patients were female (MGF *n* = 10, 58.8%; GFF *n* = 5, 41.7%). The mean age at the time of surgery was 58.5 ± 10.68 in the MGF group and 66.2 ± 9.2 in the GFF group (*p* = 0.61), with a mean BMI of 23.1 kg/m^2^ ± 4.7 in the MGF- and a mean BMI of 23.6 kg/m^2^ ± 2.7 in the GFF group (*p* = 0.94). Mean ASA score was 2.75 ± 0.43 in the MGF group and 2.58 ± 0.64 in the GFF group (*p* = 0.82). Hypertension (*n* = 8), coronary heart disease (*n* = 8), and smoking (*n* = 8) were the most frequent comorbidities, followed by diabetes (*n* = 6), malignancies other than anorectal (*n* = 5), thyroid disorders (*n* = 5), chronic inflammatory bowel disease (*n* = 5), atrial fibrillation (*n* = 4), pulmonary embolism (*n* = 4), chronic liver disease (*n* = 4), and obesity (*n* = 1). Tumor histology revealed an anal squamous cell carcinoma in six patients in the MGF group (35.3%) and in four patients in the GFF group (33.3%). Rectal adenocarcinomas were seen in 11 patients in the MGF group (64.7%) and in seven patients (58.3%) in the GFF group. One patient of the GFF group (8.3%) was diagnosed with a proctodeal gland carcinoma. Primary disease was diagnosed in just 10 out of 29 cases (*n* = 6 in the MGF group, 35.3%; *n* = 4 in the GFF group, 33.3%). Of those, six (*n* = 4 in the MGF group, 66.7% and *n* = 2 in the GFF group, 50.0%) were additionally treated by radiotherapy and chemotherapy; one patient of the GFF group received chemotherapy alone. In the cases presenting with a recurrent tumor (*n* = 11 in the MGF group, 64.7%; *n* = 8 in the GFF group 66.7%) 13 patients (*n* = 8 in the MGF group, 72.7%; *n* = 5 in the GFF group, 66.6%) were preoperatively treated with radiotherapy and chemotherapy; one patient of the MGF group (9.1%) received neoadjuvant chemotherapy alone. In the MGF group, 11 patients received PE (64.7%) and six patients received APR (35.3%). In the GFF group 11 patients received APR (91.7%) and only one patient received PE (83.3%). The choice of oncosurgical procedure led to no significant difference in the frequency of bilateral or unilateral MGF/GFF for defect reconstruction (*p* = 0.6437 in the MGF group and *p* = 0.3333 in the GFF group, Fisher's exact test). In all but four cases, microscopic complete tumor resection was achieved (R0; MGF group: *n* = 14, 82.35%; GFF group: *n* = 11, 91.67%). In cases of perineal herniation, omentoplasty was used as first choice for stabilization. In cases where neither vesicopexy nor uteropexy were feasible as second choice options, a resorbable mesh was utilized for reconstruction. In our study, omentoplasty was conducted in a total of 13 cases (MGF group: *n* = 4, 23.53%; GFF group: *n* = 9, 75%), a vesicopexy in two cases (MGF group: *n* = 1, 5.88%; GFF group: *n* = 1, 8.33%) and a mesh in 11 cases (MGF group: *n* = 7, 41.18%; GFF group: *n* = 4, 33.33%; Tables 1, 2).

**Table 1:** Demographic and oncosurgical data of the MGF group.

**Pat.- no**.	**Age**	**Sex**	**BMI**	**Comor-bidities (ASA)**	**Indication**	**Re-current disease**	**Stage**	**Pre-OP**		**Oncosurgical Proc**.
								**RT**	**CT**	
1	69	M	24	4	Rectal AC	+	ypT3,pN0,L0,V0,Pn0.R0	+	+	PE, OP, M
2	67	F	26	3	Rectal AC	+	rpT4b, pN1,L0,V0,Pn0.R0	+	+	PE, OP
3	52	M	20	3	Rectal AC	-	ypT3,pN0,L0,V0,Pn0.R0	+	+	APR
4	61	F	20	3	Rectal AC	+	ypT3,pN0,L0,V0,Pn0.R0	+	+	APR, OP
5	74	M	28	2	Rectal AC	+	rpT4,pN1,L0, V0,Pn1.R0	-	+	PE, OP
6	69	F	26	3	Anal SCC	+	rpT2,pN1,L0,V0.Pn1.R0	+	+	PE, M
7	*51*	*F*	*20*	3	Rectal AC	+	pT4,pN1,L0,V0,Pn0.R0	+	+	PE
8	*48*	*F*	*24*	3	Rectal AC	-	pT4,N2,L0,V0,Pn0.R0	-	-	APR, VP
9	*63*	*F*	*22*	3	Rectal AC	+	pT4,pN1,L0,V0,Pn1.R0	-	-	APR
10	50	M	16	3	Anal SCC	-	ypT4,pN0,L0,V1,Pn1.R1	+	+	PE, M
11	70	M	21	3	Rectal AC	-	ypT4,pN1,L0,V1,Pn1.R0	+	+	PE, M
12	36	F	16	3	Anal SCC	+	pT4,N2.L1V1,Pn1.R0	+	+	PE, M
13	59	F	20	2	Anal SCC	+	pT3,pN1, L1,V1,Pn0.R1	+	+	PE, M
14	66	M	20	3	Anal SCC	+	pT4b,pN1,L1,V0,Pn1.R1	+	+	PE
15	54	F	29	3	Anal SCC	+	rpT2,pN1,L0,V0.Pn0.R0	+	+	APR
16	64	F	25	2	Rectal AC	-	ypT3,pN0,L0,V0,Pn0.R0	-	-	PE, M
17	43	M	35	2	Rectal AC	-	rpT4b, pN1,L0,V0,Pn0.R0	+	+	APR

*M, male; F, female; BMI, body mass index in kg/m^2^; ASA, American Society of Anesthesiologists Physical Status Classification System; AC, adenocarcinoma; SCC, squamous cell carcinoma; RT, radiotherapy; CT, chemotherapy; APR, abdominoperineal resection of the rectum, PE, pelvic exenteration; OP, omentoplasty; VP, vesicopexy; M, mesh*.

**Table 2:** Demographic and oncosurgical data of the GFF group.

**Pat.- no**.	**Age**	**Sex**	**BMI**	**Comor-bidities (ASA)**	**Indication**	**Re-current disease**	**Stage**	**Pre-OP**		**Oncosurgical Proc**.
								**RT**	**CT**	
1	74	M	27	2	Rectal AC	+	pT4,pN1, L0,V0,Pn0.R0	+	+	APR, OP
2	52	M	27	2	Rectal AC	+	ypT3,pN0,L0,V0,Pn0.R0	+	+	APR, OP
3	66	F	24	3	Rectal AC	-	pT3,pN0,L1,V0,Pn1.R0	-	+	APR, OP
4	68	F	25	2	Rectal AC	+	ypT2,pN0,L0,V0,Pn0.R0	+	+	EALPE, OP
5	73	M	22	3	Rectal AC	-	pT4b, pN0,L1,V0,Pn1.R0	-	+	APR, OP, M
6	58	M	27	2	Rectal AC	-	ypT1,pN0,L0,V0,Pn0.R0	+	+	APR, M
7	*72*	*M*	*21*	3	Rectal AC	+	rpT3,pN0,L0,V0,Pn0. R0	-	-	APR, VP
8	*62*	*M*	*19*	4	Anal SCC	+	ypT3,pN0,L0,V0,Pn1.R0	+	+	APR, OP
9	*69*	*F*	*25*	3	Anal SCC	+	pT4,pN0, L1,V1,Pn1. R1	-	-	APR, OP
10	49	F	20	3	Anal SCC	+	pT4,pN0,L1V1,Pn0. R0	+	+	APR, M
11	83	M	22	2	Anal SCC	+	ypT3,pN0,L0,V0,Pn0.R0	-	-	APR, OP, M
12	68	F	24	2	Proctideal gland C	-	ypT3,pN0,L0,V0,Pn0.R0	+	+	EP, OP

*M, male; F, female; BMI, body mass index in kg/m^2^; ASA, American Society of Anesthesiologists Physical Status Classification System; AC, adenocarcinoma; SCC, squamous cell carcinoma; C, carcinoma; RT, radiotherapy; CT, chemotherapy; APR, abdominoperineal resection of the rectum, PE, pelvic exenteration; OP, omentoplasty; VP, vesicopexy; M, mesh*.

Concerning defect coverage and obliteration of dead space, 10 patients received unilateral flaps in the MGF group (58.82%). Among those receiving GF-flaps, only four patients (33.33%) were treated with unilateral flaps (*p* = 0.2635, Fisher's exact test). Taken together, a close majority of 15 patients was treated with bilateral flap coverage. Mean operation time of flap coverage for unilateral flaps was 105 ± 9 min in the MGF group and 70 ± 13 min in the GFF group (*p* = 0.0497). For bilateral flaps, flap coverage took 163 ± 11 min in the MGF group and 107 ± 14 min in the GFF group (*p* = 0.0077).

In 11 patients, we saw no complication (37.93%) at all. According to the Clavien-Dindo classification for surgical complications, the were 4 grade II, 3 type IIIa, 3 type IIIb, and one type 4a complication among MGFs. In the GFF group, one type II, 2 type IIIa, and 4 type IIIb complications were observed. There was no significant difference between the two groups. Type II complications were postoperative infections that could be treated with antibiotic therapy. Type IIIa complications included wound healing disorders of the donor site or defect site and local infections or seroma formation resulting in bed site debridement or drainage. Type IIIb complications included wound dehiscence and partial flap loss (<30%) that had to be treated by debridement, vacuum assisted closure (VAC) or secondary suture under general anesthesia. There was one grade IVa complication (intraoperative ventricular fibrillation) that resulted in a staged though successful defect coverage in the MGF group. We saw no breakdown of enteric anastomoses, no formation of vascular or visceral fistulae, and no instances of deep pelvic abscess formation. The time from reconstruction to discharge was 23 ± 4.7 days for MGFs and 24 ± 9.7 days for GFFs (*p* = 0.9002). Regarding both groups, we found no significant difference in the time to discharge between patients with complications of any grade and those who were unaffected (*p* = 0.9190) (Tables 3, 4). Analyzation of relevant risk factors (gender, age, BMI, comorbidities, preoperative radio-/chemotherapy, primary, or recurrent disease, and number of flaps) for complications or delayed discharge by univariate analysis revealed no single significant factor. With a mean follow-up of 17 ± 9.20 months among MGFs and 16 ± 8.88 months among GFFs (*p* = 0.9203), flap-related complications were documented. In the GFF group 5 (29.41%) patients had pain under mobilization and 2 (11.77%) patients complained about pain at the donor site when sitting within the first 30 postoperative days. Among GFFs, 3 (25.0%) patients felt pain under mobilization and 7 (58.33%) patients complained about pain at the donor site when sitting. Thus, significantly more patients felt sitting-related pain at the donor site in the GFF group (*p* = 0.0104, Fisher's exact test). No long-lasting (>30 days) flap related disability was documented in both groups.

**Table 3:** Reconstructive and postoperative data of the MGF group.

**Pat.- no**.	**Re-constr. Proc**.	**Time for defect coverage (min)**	**Complications**			**Post-op stay (days)**	**Follow-up (months)**
			**CD-Class**.	**Type**	**Management**		
1	Bilateral	191	Iva	Intraoperative ventricular fibrillation	Reanimation, staged coverage	33	25
2	Unilateral	91	-			21	5
3	Unilateral	84	II	Postoperative infection	Antibiotic therapy	25	16
4	Unilateral	131	IIIa	Wound healing disorder (defect site)	Debridement, VAC	26	32
5	Bilateral	209	IIIa	Seroma formation (donor site)	Puncture	20	7
6	Bilateral	125	IIIb	Wound dehiscence (defect site)	Debridement, VAC	28	6
7	Unilateral	97	IIIa	Local Infection (defect site)	Drainage	22	13
8	Unilateral	131	-			24	12
9	Bilateral	121	-			16	25
10	Unilateral	97	IIIb	Wound healing disorder (defect site)	Debridement, VAC	21	21
11	Bilateral	177	II	Postoperative infection	Antibiotic therapy	19	15
12	Unilateral	101	II	Postoperative infection	Antibiotic therapy	23	24
13	Unilateral	86	-			25	3
14	Unilateral	122	-			13	34
15	Bilateral	152	IIIb	Partial flap loss (<30%)	Debridement, flap repositiong	28	18
16	Bilateral	162	-			21	27
17	Unilateral	116	-			19	12

*Proc, Procedure; min, minutes; CD-Class, Clavien-Dindo classification; VAC, vacuum assisted closure; Post-op stay time from reconstruction to discharge in days*.

**Table 4:** Reconstructive and postoperative data of the GFF group.

**Pat.- no**.	**Re-constr. Proc**.	**Time for defect coverage (min)**	**Complications**			**Post-op stay (days)**	**Follow-up (months)**
			**CD-Class**.	**Type**	**Management**		
1	Unilateral	53	-			11	4
2	Bilateral	103	II	Postoperative infection	Antibiotic therapy	24	28
3	Unilateral	110	-			33	16
4	Bilateral	98	II	Postoperative infection	Antibiotic therapy	21	12
5	Bilateral	187	IIIb	Wound dehiscence (defect site)	Debridement, VAC	21	21
6	Bilateral	168	-			22	12
7	Bilateral	125	IIIb	Wound dehiscence (defect site)	Debridement, VAC	25	6
8	Bilateral	97	-			14	9
9	Bilateral	106	IIIb	Wound dehiscence (donor site)	Debridement, secondray suture	28	31
10	Unilateral	87	IIIa	Local abscess formation	Drainage	13	28
11	Bilateral	135	IIIb	Wound healing disorder (defect site)	Debridement, secondary suture	25	7
12	Unilateral	75	IIIa	Wound dehiscence (defect site)	Debridement	49	14

*Proc, Procedure; min, minutes; CD-Class, Clavien-Dindo classification; VAC, vacuum assisted closure; Post-op stay time from reconstruction to discharge in days*.

## Discussion

Abdominoperineal resections create a wound that is intrinsically poor at healing due to the location, frequent bacterial contamination, and dead space prone to fluid collection (5). Preoperative chemoradiation, associated comorbidities, and pressure created by sitting upright complicate the healing process. As such, wound complication rate of up to 60% are reported in the literature (5, 8, 18, 19). A flap-based wound closure is the idea to obliterate dead space and to recruit well-vascularized tissue into the irradiated wound bed, thereby improving blood-flow, antibiotic delivery and healing (11, 20–22). Several series have demonstrated the beneficial effect of immediate defect reconstruction with regional flaps when compared with primary closure however, the exact indications for flap closure vs. direct closure are still debated (11, 20, 22–25). In the past, pelvic defects have commonly been reconstructed with vertical rectus abdominis myocutaneous (VRAM) flaps, as the large-volume bulk effectively obliterates pelvic dead space (3, 5, 21, 26). However, harvest of the rectus abdominis muscle can result in weakening of the abdominal wall, abdominal bulge or hernia, mesh-related complications, if a mesh is required, and in many cases the flap may be unavailable because of pre-existing abdominal scars or the need for colostomy/urostomy or both (5, 27–29).

We here compare two well-described concurrent techniques that are used in our department. The gracilis muscle is the most superficial adductor of the thigh and harvest of the myocutaneous flap paddle results in minimal functional deficit (5, 30). To date, there are conflicting reports in terms of reliability of the flap for pelvic reconstruction as high (31) and very low complication rates (32) have been reported. This warrants further investigation as addressed in this study. Regional alternatives to muscle-based flaps represent perforator-based flaps of the internal pudendal artery (terminal branch of the internal iliac artery) (14, 17, 33). Though the initial description of the gluteal fold flap dates back to 1996 (14), reports of its use in anorectal resection for malignancy are relatively sparse (2, 13, 34). This may reflect the uncertainty about the residual blood supply following extensive pelvic dissection or the habitus-dependent limitation of tissue bulk to fill dead space in the pelvis. Among others, the MGF and the GFF are well-described alternatives to the VRAM flap in the literature. However, there is to date no comparative outcome study that compares the flaps types in terms of clinical outcome parameters.

This study illustrates the limitations and benefits of the muscle based MGF and the perforator based GFF in a comparable patient collective. In a close majority of our patients, defect coverage with obliteration of dead space could only be achieved through bilateral flap elevation. There was no significant difference between MGFs and GFFs, which allows the conclusion that mobilization of tissue bulk is comparable for both flaps even though substantial inter-individual differences in the distribution of subcutaneous body fat in the region of the thigh and gluteal fold could be observed. In this respect, a BMI >25 did not increase the chance for unilateral flap coverage. The obliteration of dead space is effective with single VRAM flaps, however, as defect size reduces; the ability to fit a large VRAM (especially in obese patients with thick abdominal tissue) gets more difficult (5). Even if bilateral myocutaneous gracilis or gluteal fold mobilization is needed, morbidity to the patient is reduced compared to VRAM flaps (5).

Skin perfusion problems, resulting in skin necrosis in the distal part because of inconsistent perforator blood supply is a well-documented complication of the MGF (6, 35). Anatomic studies of the proximal gracilis pedicle illustrated both septocutaneous and myocutaneous perforators traveling in a transverse direction, suggesting the skin island for the MGF should be redesigned in a horizontal fashion (6, 36). To date, several authors prefer the horizontal skin island design (transverse myocutaneous gracilis flap, TMG flap) and achieve flap dimensions that are comparable to the vertical flap design (37). Further developments included a bilobed design of the MGF for perineal reconstruction (6). Studies reexamining the perforator anatomy and cutaneous vascular supply of MGFs found a variable quantity of gracilis perforators perfuse a nearly circular shaped angiosome centered over the proximal muscle (6, 38, 39). A circular design of the skin island would therefore be preferable, though unacceptable in terms of donor site mortality.

**Figure 1:**
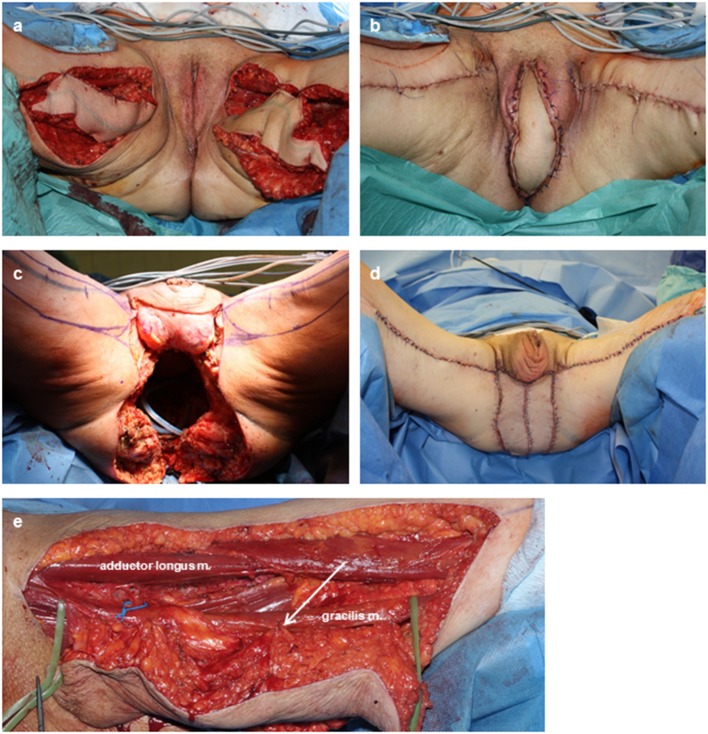
Intra- and postoperative documentation of MGFs. **(a,b)** Bilateral defect coverage after PE with vulvectomy in a case of advanced recurrent rectal AC. **(c,d)** Bilateral defect coverage after extended PE with amputation of the penis and testecomy in a case of recurrent anal SCC. The extended cutaneous defect resulted in a cutaneous coverage through both skin islands. **(e)** Flap elevation. Sutures (arrow) between the muscle and its skin paddle prevented tension forces to the perforators. The main vascular pedicle (loop) is freed to its junction for maximal mobility of the flap.

In our experience, the skin island of the MGF is reliable as long as it is centered over the superior two-thirds of the muscle. This results in flap dimensions that are comparable to the TMG flap design. Suturing the skin island to the gracilis muscle with resorbable sutures during flap elevation is effective taking traction forces from the perforators (Figure 1). Inspection of the skin island in the distal part before flap insertion is mandatory to identify and remove insufficiently perfused cutaneous and subcutaneous tissue. Alternatively, indocyanine green (ICG) imaging can be performed to evaluate tissue perfusion intraoperatively and may be superior to sole inspection of the skin (40). Under those measures, the MGF is a reliable flap and flap necrosis is reduced to a minimum. Here, we saw only one partial flap necrosis (<30%) in the MGF group that could be attributed to perfusion problems and resulted in operative debridement and repositioning of the flap. The rates of partial flap loss among MGF (6%) are comparable to those that have been reported for TMG flaps (Kaartinen et al. 6%; Kiiski et al. 4%) (37, 41).

**Figure 2:**
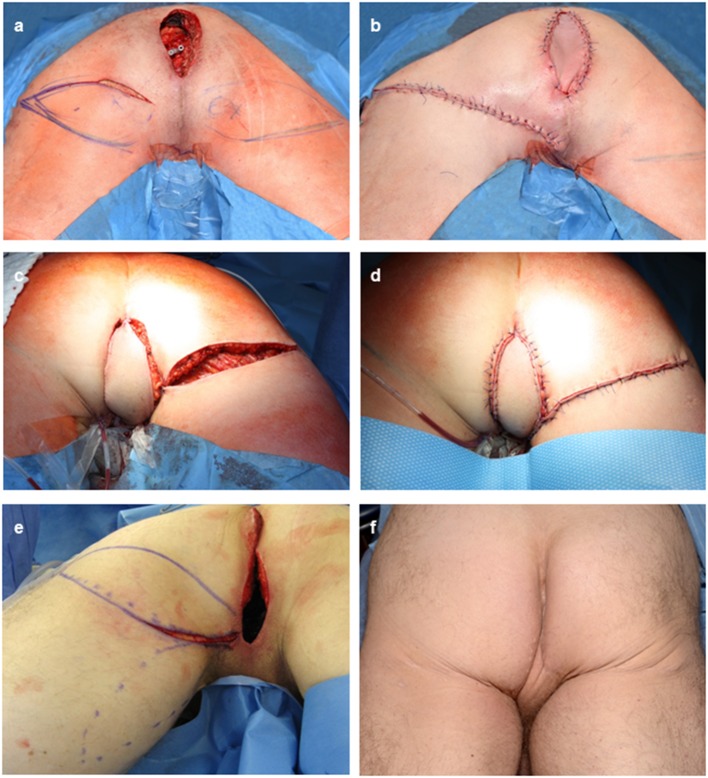
Intra- and postoperative documentation of GFFs. **(a)** Perineal defect after APR for recurrent rectal AC with the potential for a bilateral flap desgin. **(b)** flap insertion after tunneling of the GFF and primary closure of the defect. **(c,d)** Right sided GFF without skin bridge to the perineal defect after APR for a recurrent anal SCC. **(e)** Intraoperative markings of either usage of the left sided MGF or GFF. Here, the GFF was used. **(f)** Posoperative result after bilateral GFF.

Elevation of gluteal fold perforator flaps has been described in a sub-fascial and epi-fascial plane with or without strict identification of the pedicle, the latter with the idea to prevent pedicle torsion (Figure 2) (7, 14). We here avoided to skeletonize the pedicle in order to overcome previously described venous congestion of the GFF (42) which also contributed to minimize the operation time of the reconstructive part. The flap was designed to contain the Doppler signal in the rotation axis (type I-1 pattern according to Hashimoto et al.). The propeller design allowed easy movement of the entire flap and avoided dog ear formation around the flap that can occur with larger transposition flaps (type I-2 pattern according to Hashimoto et al.). Defect coverage was significantly faster with GFFs compared to MGFs, either uni- or bilaterally. In this respect, the GFF is superior to the MGF as it reduces the time for the patient in surgery. However, planning for GFFs including Doppler examination is more time consuming than for MGF. Also, intraoperative confirmation of the preoperative Doppler examination is advisable, as gluteal perforators can be weakened through extensive tumor resection. Elevation of the GFF is also possible in lithotomy or Lloyd Davis positioning, however it is significantly more complex. Conversely, Jackknife positioning complicates elevation of gracilis based flaps, thus prolonging operation time.

An equivalent surgical complication rate in patients receiving MGFs and those receiving GFFs is a significant finding of our study. Most of the patients had complications (62.07%) however, the vast majority was of minor degree and treatable with minimal intervention. There was no complete flap loss and complication rates of GFFs are in line with those reported in the literature (2, 7, 13, 42). Different experience is reported on MGFs for perineal defect coverage, complicating the assessment of our own results. Chong et al. (32) reported lower complication rates whereas others (31) saw distinctly higher complication rates with myocutaneous gracilis flaps. Our report clearly demonstrates that the GFF is not superior to the MGF, as reported by others (13). The previously reported limitation of the MGF in terms of tissue bulk and mobility can be overcome by generous planning of flap dimensions, complete dissection of the vascular pedicle and bilateral flap elevation if necessary.

In either using the MGF or the GFF for defect coverage uni- or bilaterally, discharge was not significantly influenced by complications. Besides, we found no independent risk factor among patients for complications or time to discharge, although this may be due to the small number of cases in our series. Morbidity of MGFs and GFFs is low, even when raised bilaterally. No long-lasting flap related disability was documented in both groups which is in contract to the VRAM flap, where rates of incisional hernia have been reported to be as high as 10% after flap harvest (27–29). Sitting associated pain is an issue among patients after gluteal fold flap harvest. This is well-explained by the postoperative position of the scar. However, when clearly communicated preoperatively, this is well-tolerated by most patients as a temporary discomfort.

Although no complication could be attributed to the utilization of a mesh, we try to avoid this technique and rather use the greater omentum for the closure of the pelvic entrance. Only sometimes, fully resorbable Polyglactin mesh had to be used in order to prevent a small bowel herniation into the deep pelvic at early postoperative stages. We are strongly opposed to non-resorbable or synthetic meshes in the pelvis, especially because the surgeries described here are “clean-contaminated” at best.

This study compares two alternative techniques for perineal defect reconstruction with the intention to provide a comparable patient collective and a comparable patient number. Concurrent techniques such as IGAP advancement flap or the posterior thigh flap are therefore not included (9, 10, 43).

## Conclusion

**Figure 3:**
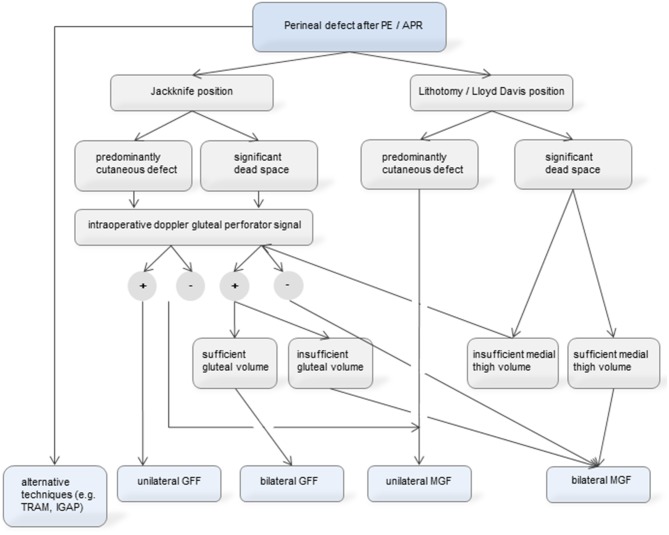
Proposed algorithm for decision-making in perineal defect reconstruction through MGF and GFF. As MGFs and GFFs are equally effective and safe, the decision can be based on individual factors. These are patient positioning, gluteal perforator quality and body habitus (distribution of subcutaneous fat and skin laxity at the thigh and the gluteal fold). The algorithm focuses on the MGF and GFF, we however emphasize that alternative techniques such as TRAM flap or IGAP flap can be used and are not included in the decision making presented herein.

Our study demonstrates the safety and efficacy of gracilis based myocutaneous flaps as well as gluteal fold flaps to reconstruct perineal defects secondary the abdominoperineal excision of the rectum and pelvic exenteration. The overall complication rate is equivalent for both types of flaps. Beneficial effects of each flap such as operation time and postoperative rehabilitation will even out at the end, so that we propose the equal application. Decision-making should be based on individual factors such as body habitus (distribution of subcutaneous fat and skin laxity at the thigh and the gluteal fold), intraoperative patient positioning (dependent on colorectal surgeon preference), and gluteal perforator distribution and quality (Figure 3).

## Data Availability Statement

The datasets generated for this study are available on request to the corresponding author.

## Ethics Statement

The studies involving human participants were reviewed and approved by Freiburg Ethics Committee Engelberger Strasse 21 79106 Freiburg Germany. The patients/participants provided their written informed consent to participate in this study. Written informed consent was obtained from the individual(s) for the publication of any potentially identifiable images or data included in this article.

## Author Contributions

SE and JT conceived of the presented idea. JT and JW developed the theory and performed the computations. HN, PM, SE, and SF-F verified the analytical methods. GS and SE encouraged JT to investigate the therapy-related data and supervised the findings of this work. All authors discussed the results and contributed to the final manuscript and gave their final approval of the version to be submitted.

## Conflict of Interest

The authors declare that the research was conducted in the absence of any commercial or financial relationships that could be construed as a potential conflict of interest.
